# The interaction of ethnicity and deprivation on COVID-19 mortality risk: a retrospective ecological study

**DOI:** 10.1038/s41598-021-91076-8

**Published:** 2021-06-02

**Authors:** Kausik Chaudhuri, Anindita Chakrabarti, Jose Martin Lima, Joht Singh Chandan, Siddhartha Bandyopadhyay

**Affiliations:** 1 Economics Division, Leeds University Business School, Leeds, LS2 9JT UK; 2 Institute of Applied Health Research, University of Birmingham, Birmingham, B152TT UK; 3 Department of Economics, Birmingham Business School and Centre for Crime Justice and Policing, University of Birmingham, Birmingham, B15 2TT UK

**Keywords:** Infectious diseases, Scientific data, Statistics

## Abstract

Black, Asian and Minority Ethnic (BAME) populations are at an increased risk of developing COVID-19 and consequentially more severe outcomes compared to White populations. The aim of this study was to quantify how much of the disproportionate disease burden can be attributed to ethnicity and deprivation as well as its interaction. An ecological study was conducted using data derived from the Office for National Statistics data at a Local Authority District (LAD) level in England between 1st March and 17th April 2020. The primary analysis examined how age adjusted COVID-19 mortality depends on ethnicity, deprivation, and the interaction between the two using linear regression. The secondary analysis using spatial regression methods allowed for the quantification of the extent of LAD spillover effect of COVID-19 mortality. We find that in LADs with the highest deprivation quartile, where there is a 1 percentage point increase in “Black-African (regression coefficient 2.86; 95% CI 1.08–4.64)”, “Black-Caribbean (9.66: 95% CI 5.25–14.06)” and “Bangladeshi (1.95: 95% CI 1.14–2.76)” communities, there is a significantly higher age-adjusted COVID-19 mortality compared to respective control populations. In addition, the spatial regression results indicated positive significant correlation between the age-adjusted mortality in one LAD and the age-adjusted mortality in a neighbouring LAD, suggesting a spillover effect. Our results suggest targeted public health measures to support those who are deprived and belong to BAME communities as well as to encourage restricted movement between different localities to limit disease propagation.

## Introduction

The COVID-19 pandemic, caused by the Severe Acute Respiratory Syndrome Coronavirus-2 (SARS-CoV-2) was originally declared a public health emergency on January 30th 2020^1^, and has since led to the deaths of over 700,000 individuals globally^2^. The first recorded case in England was on the 29th January 2020, following which England has become one of most globally affected countries in terms of case numbers and mortality^2,3^.

The United Kingdom (UK) is an ethnically diverse country, and as the pandemic has progressed we find that the risk of both COVID-19 susceptibility and mortality varies with ethnicity. Medical records from primary and secondary care as well as death certificates examined by the Office for National Statistics (ONS), Public Health England (PHE) and other academics make it clear that those from Black, Asian and Minority Ethnic (BAME) communities are at the highest risk of both COVID-19 susceptibility and associated mortality^4–6^. These findings have been replicated internationally and raise concern for urgent public health action to identify the cause for such a correlation^7,8^.

However, an important point to note is that within the UK and abroad, those from BAME communities are more likely to live in deprived areas which may in turn increase their risk of health inequalities when compared to the reference White population in the UK^9^. Existing data taken by the ONS and PHE examined the impact of age, gender and deprivation separately on COVID-19 mortality rates but did not examine the interaction between ethnicity and deprivation and how this may affect such risk^4,5^. More recent studies examining the impact of ethnicity on COVID-19 using intensive care unit data and hospital data clearly raise the need for examining the association with socio-economic characteristics in more detail and also for the urgent need to identify cultural risk factors which may be susceptible for intervention^10,11^.

When considering the effect between ethnicity and deprivation, it must also be important to analyse “neighbourhood effects,” which as seen even in the Global Burdens of Disease work have a substantial effect on population health^12,13^. Neighbourhood effect is defined as the impact from living in a particular locality^14^, in this case we can think of it as a LAD. A large literature exists that suggest that one’s neighbourhood can affect individual outcomes, including health^15^. Spillovers refer to effects percolating from one LAD to a contiguous one. Therefore, for an effective analysis examining the interaction between ethnicity, deprivation and COVID-19 risk we must consider the impact of the neighbourhood effect as well as spillover effects between neighbouring regions. Not considering these spillover effects would potentially underestimate the full effect of interaction between ethnicity, deprivation and COVID mortality. Although some work has started to examine spatial distribution of COVID-19 even within the UK, to our knowledge no studies have examined spatial distribution of infection rates between Local Authorities Districts (LAD) in the UK which would give an indication of how COVID-19 is being transmitted due to social interaction and local travel^16–18^.

Therefore, the first aim of this study was to examine the interaction between ethnicity and deprivation when associating ethnicity to COVID-19 mortality. In addition, we aimed to examine spillover effects between neighbouring LADs in England.

## Methods

### Study design and participants

In this study we used open source LAD (a subnational division of England used for the purposes of local government) level data from England to undertake a cross sectional analysis of our variables of interest. There are 317 LADs in England, and we used data from 315 of them excluding the City of London and the Isle of Sicily which were dropped due to missing observations. The primary focus of this study was to analyse the disparity in COVID-19 deaths for individuals belonging to BAME groups vis-à-vis the White population (while neither of these categories are homogenous, we use sub-groups as used by the Census)^19^. Secondly, we assessed the importance of the index of multiple deprivation (IMD), in explaining the age adjusted mortality across the different local areas in England after we control for ethnicity. Finally, we quantified the extent of a spillover effect of COVID-19 disease burden that helped us to assess the impact of social-distancing measures introduced by the government.

### Data sources and definitions

The data are taken from different sources. Our dependent variable is the age standardised death rate data for all persons at LADs in England is taken from the ONS across 315 LADs from 1 March to 17 April 2020^20^. This is defined as the age adjusted mortality per 100,000 of the population of the LAD. We took into account LAD boundary changes which occurred in April 2019, for example Bournemouth, Poole and Christchurch combined as one LAD. The average score for the index of multiple deprivation, ethnicity data and educational attainment used were taken from the Office for National Statistics^19,21^. Data on density of population expressed as total population per hectare was taken from Census 2011, Table PHP01. A-level educational qualification (proportion with GCE A level or equivalent—aged 16–64) were taken from https://www.nomisweb.co.uk/query/construct/components/stdSearch.asp?menuopt=7&subcomp=131.

We classified ethnicity using the broad headings used in the 2011 Census^22^. Our dependent variable and independent variables are described in Table 1.

**Table 1:** Characteristics of LADs: summary statistics.

Variable	Overall^b^ Mean (SD)	Quartile 1 Mean (SD)	Quartile 2 Mean (SD)	Quartile 3 Mean (SD)	Quartile 4 Mean (SD)
Age-adjusted Mortality (per 100,000 people)	35.501 (24.858)	27.727 (11.256)	32.057 (23.603)	35.009 (25.656)	47.210 (32.245)
IMD (Average score)	19.641 (7.986)	10.538 (1.887)	15.680 (1.483)	21.703 (2.030)	30.643 (4.579)
Black-African^a^	1.311 (2.420)	0.451 (0.341)	1.086 (1.608)	1.551 (2.598)	2.155 (3.552)
Black-Caribbean^a^	0.739 (1.518)	0.270 (0.272)	0.557 (0.855)	0.880 (1.746)	1.249 (2.218)
Black-Other^a^	0.340 (0.756)	0.101 (0.077)	0.244 (0.395)	0.416 (0.827)	0.601 (1.041)
Chinese^a^	0.600 (0.585)	0.503 (0.327)	0.624 (0.680)	0.544 (0.571)	0.730 (0.674)
Bangladeshi^a^	0.592 (2.051)	0.183 (0.230)	0.345 (0.579)	0.446 (0.793)	1.393 (3.935)
Indian^a^	2.051 (3.638)	1.510 (2.287)	2.002 (3.836)	1.991 (3.301)	2.702 (4.677)
Pakistani^a^	1.389 (2.894)	0.589 (1.179)	0.736 (1.638)	1.291 (2.718)	2.939 (4.341)
Asian-Other^a^	1.252 (1.671)	0.961 (1.066)	1.393 (2.129)	1.330 (1.814)	1.323 (1.482)
Mixed^a^	1.893 (1.382)	1.566 (0.665)	1.741 (1.150)	1.979 (1.599)	2.288 (1.759)
Arab^a^	0.294 (0.616)	0.158 (0.217)	0.221 (0.313)	0.393 (1.025)	0.404 (0.538)
Other Ethnic Group^a^	0.462 (0.708)	0.263 (0.230)	0.382 (0.536)	0.529 (0.839)	0.674 (0.941)
White^a^	89.076 (12.996)	93.445 (5.099)	90.669 (11.717)	88.649 (13.683)	83.542 (16.687)
Observations	316	79	79	79	79

Quartile 1–4 denotes the quartiles based on index of multiple deprivation. Quartile 1 is least deprived and Quartile 4 is the most deprived.

^a^Denotes in terms of percentage of total population in the LAD.

^b^Overall indicates for the whole of England.

The dependent variable is the age adjusted COVID-19 death rate per 100,000 in the population. The ‘Quartile Dummy_i_ (j = 1, 2, 3,4)’ presents the quartile dummies for the index of multiple socio-economic deprivation. We use ‘Quartile 1 Dummy’ representing the bottom 25% (least deprived) as the base. In addition, two other explanatory variables were incorporated to capture population per square hectare kilometre within each LAD (i.e. $pop-density$) and education status within a LAD (*edu-status*) measured in terms of proportion of individuals within LAD who have completed A-level. Each ethnicity variable represented the % of people with that ethnicity in that LAD. Our categorisation of the ethnicity variable departs from other previous studies by disaggregating within broad ethnic groups^10^. Instead of using Black as a whole, we divide ‘Black’ into ‘Black-African’, ‘Black-Caribbean’ and ‘Black-Others’. For the Asian ethnicity, we use the following categories: ‘Bangladeshi’, ‘Indian’, ‘Pakistani’, ‘Arab’, ‘Chinese’ and ‘Asian-Other’. The ‘Mixed’ category is a combination of ‘Mixed White & Asian’, ‘Mixed White & Black African’, ‘Mixed White and Black Caribbean’ and ‘Mixed Other’. Along with the above, we also have ‘Other Ethnic group’ and the excluded group is the ‘White’ community which hence serves as the reference category.

### Statistical analysis

Two types of statistical analysis were performed.

A: An OLS (ordinary least squares) regression model was estimated to see the impact of ethnicity, particularly the interaction of ethnicity with economic deprivation in explaining the mortality differential across the LADs while controlling for population density and education status that may also affect mortality.

We note that the sum of the ethnic population percentages in each LAD equals 100. Therefore, we have excluded the ‘White’ community, which acts as the reference category, to eliminate the collinearity problem. The estimated equation takes the following form:

1 $$ \begin{aligned}{\mathrm{COVID}}_{\mathrm{i}}&=\mathrm{\alpha }+\sum_{\mathrm{j}=2}^{4}{{\upbeta }_{\mathrm{j}}\mathrm{Quartile Dummy}}_{\mathrm{i}}+{\upbeta }_{5}{\mathrm{Bangladeshi}}_{\mathrm{i}}+{\upbeta }_{6}{\mathrm{Indian}}_{\mathrm{i}}\\ &\quad+{\upbeta }_{7}{\mathrm{Pakistani}}_{\mathrm{i}}+{{\upbeta }_{8}{\mathrm{Arab}}_{\mathrm{i}}+\upbeta }_{9}{\mathrm{Chinese}}_{\mathrm{i}}+{\upbeta }_{10}{\mathrm{Asian}-\mathrm{Other}}_{\mathrm{i}}{+\upbeta }_{11}\mathrm{Black}-{\mathrm{African}}_{\mathrm{i}}\\ &\quad+{\upbeta }_{12}\mathrm{Black}-{\mathrm{Caribbean}}_{\mathrm{i}}+{\upbeta }_{13}\mathrm{Black}-{\mathrm{Other}}_{\mathrm{i}} +{\upbeta }_{14}{\mathrm{Mixed}}_{\mathrm{i}}+{\upbeta }_{15}{\mathrm{Other}-\mathrm{Ethnic}}_{\mathrm{i}}\\ &\quad+\sum_{\mathrm{j}=2}^{4}{{\upgamma }_{\mathrm{j}}\mathrm{Quartile}}_{\mathrm{j}}*{\mathrm{Bangladeshi}}_{\mathrm{i}}+ \sum_{\mathrm{j}=2}^{4}{{\mathrm{\varnothing }}_{\mathrm{j}}\mathrm{Quartile}}_{\mathrm{j}}*{\mathrm{Indian}}_{\mathrm{i}}+\sum_{\mathrm{j}=2}^{4}{{\upmu }_{\mathrm{j}}\mathrm{Quartile}}_{\mathrm{j}}*{\mathrm{Pakistani}}_{\mathrm{i}}\\ &\quad++\sum_{\mathrm{j}=2}^{4}{{\uptau }_{\mathrm{j}}\mathrm{Quartile}}_{\mathrm{j}}*{\mathrm{Arab}}_{\mathrm{i}}+\sum_{\mathrm{j}=2}^{4}{{\mathrm{\vartheta }}_{\mathrm{j}}\mathrm{Quartile}}_{\mathrm{j}}*{\mathrm{Chinese}}_{\mathrm{i}}+\sum_{\mathrm{j}=2}^{4}{{\upeta }_{\mathrm{j}}\mathrm{Quartile}}_{\mathrm{j}}*\mathrm{Asian}-{\mathrm{Other}}_{\mathrm{i}}\\ &\quad+\sum_{\mathrm{j}=2}^{4}{{\updelta }_{\mathrm{j}}\mathrm{Quartile}}_{\mathrm{j}}*\mathrm{Black}-{\mathrm{African}}_{\mathrm{i}}+\sum_{\mathrm{j}=2}^{4}{{\uptheta }_{\mathrm{j}}\mathrm{Quartile}}_{\mathrm{j}}*\mathrm{Black}-{\mathrm{Carribean}}_{\mathrm{i}}\\ &\quad+\sum_{\mathrm{j}=2}^{4}{{\uplambda }_{\mathrm{j}}\mathrm{Quartile}}_{\mathrm{j}}*\mathrm{Black}-{\mathrm{Other}}_{\mathrm{i}}+\sum_{\mathrm{j}=2}^{4}{{\uppsi }_{\mathrm{j}}\mathrm{Quartile}}_{\mathrm{j}}*{\mathrm{Mixed}}_{\mathrm{i}}\\ &\quad+\sum_{\mathrm{j}=2}^{4}{{\upomega }_{\mathrm{j}}\mathrm{Quartile}}_{\mathrm{j}}*{\mathrm{Other}-\mathrm{Ethnic}}_{\mathrm{i}}+{\mathrm{\vartheta }{\mathrm{pop}-\mathrm{density}}_{i}+\Psi {\mathrm{edu}-\mathrm{status}}_{i }+\mathrm{u}}_{\mathrm{i}}\end{aligned} $$

where the dependent variable is the age adjusted COVID-19 death rate per 100,000 in the population in LAD i. The independent variables are as described above. The term u represents the error term.

To justify the linear specification and rule out a non-linear relationship, we performed a specification test where the predicted value and its square from the estimated OLS regression were used as explanatory variables in a separate regression with the same dependent variable. It turned out that the squared of the predicted value had a p-value of 0.416, implying that our OLS model was correctly specified. Therefore, we excluded the possibility of a non-linear relationship. To test for presence of multicollinearity, we ran a model without the interaction terms. The average variance inflation factor was 5.60 which is lower than the tolerance value of 10.

The model performance in terms of goodness of fit statistics (${R}^{2}$) is 0.782. We also performed an information matrix test for the regression model and an orthogonal decomposition into tests for heteroskedasticity, skewness, and kurtosis. The *p* value for test of heteroskedasticity, skewness, and kurtosis was 0.474, 0.991 and 0.150 respectively justifying our specified model.

As a robustness check, given the dependent variables is censored between lower-limit (minimum) and upper-limit (maximum), we ran a censored regression model. The results obtained from the censored regression model were very similar to the one reported in the paper from the linear regression.

**Figure 1:**
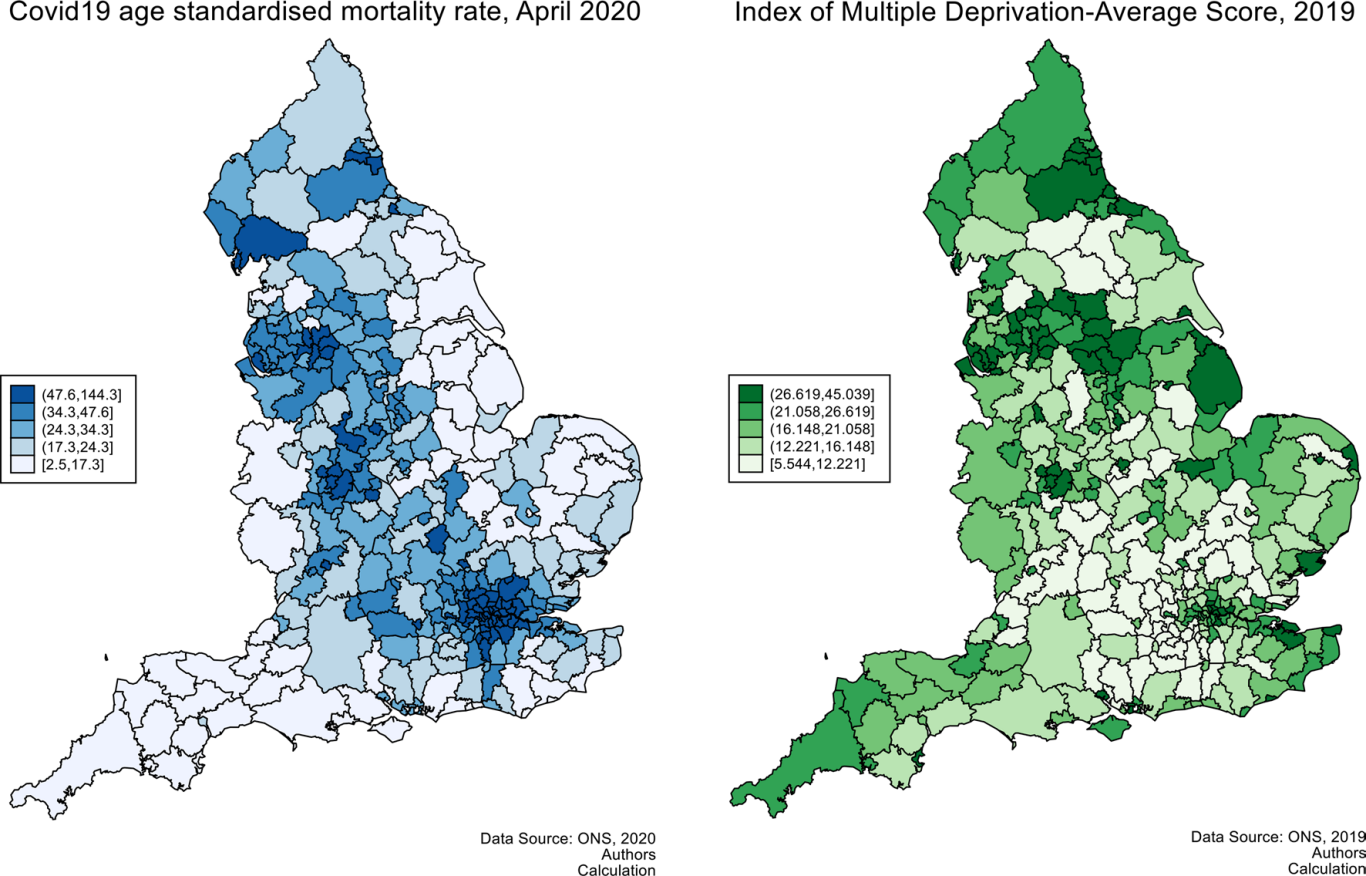
Age adjusted mortality rate and index of multiple deprivation.

B: Spatial analysis. Figure 1 displays the age-adjusted mortality rates along with index of multiple deprivations across 315 LADs in England, comprising an area with 54,706,877 inhabitants and 173,673 inhabitants per LAD on average. The colour scheme depicted in a colour gradient showed lowest to highest values of age-adjusted mortality rates (in shades of blue) and index of multiple deprivation (in shades of green).

The COVID-19 age standardised mortality rate is clustered in the London metropolitan area and its surroundings, the Midlands, the Liverpool-Manchester area, the south of the Cumbria region, and in the North East (especially Durham and Newcastle), and lower down in the South, the South West, the Yorkshire and Humber region, the East, and in the East Midlands. This picture contrasts with the existing disparities in IMD across England, where the South and the East present high levels of IMD, suggesting that the mortality rate may not be completely explained by deprivation at the local level. Other factors, like ethnic backgrounds and derived social habits, could also help to explain the mortality rate.

To investigate spillover effect from neighbouring LADs (keeping in mind the greater likelihood of social interaction, even during the lockdown, across neighbouring LADs), we undertook a spatial regression analysis using IMD and percentages of ethnic population as the explanatory variables. We allowed for spatial dependence in the age standardised mortality rate (especially given the North–South divide in England). In particular, we estimated the following equation:

2 $$ \begin{aligned}{\mathrm{COVID}}_{\mathrm{i}}&=\mathrm{\alpha }+\mathrm{\phi WCovi}{\mathrm{d}}_{\mathrm{i}}+\sum_{\mathrm{j}=2}^{4}{{\upbeta }_{\mathrm{j}}\mathrm{Quartile Dummy}}_{\mathrm{i}}+{\upbeta }_{5}{\mathrm{Bangladeshi}}_{\mathrm{i}}+{\upbeta }_{6}{\mathrm{Indian}}_{\mathrm{i}}\\ &\quad+{\upbeta }_{7}{\mathrm{Pakistani}}_{\mathrm{i}}+{{\upbeta }_{8}{\mathrm{Arab}}_{\mathrm{i}}+\upbeta }_{9}{\mathrm{Chinese}}_{\mathrm{i}}+{\upbeta }_{10}{\mathrm{Asian}-\mathrm{Other}}_{\mathrm{i}}{+\upbeta }_{11}\mathrm{Black}-{\mathrm{African}}_{\mathrm{i}}\\ &\quad+{\upbeta }_{12}\mathrm{Black}-{\mathrm{Caribbean}}_{\mathrm{i}}+{\upbeta }_{13}\mathrm{Black}-{\mathrm{Other}}_{\mathrm{i}} +{\upbeta }_{14}{\mathrm{Mixed}}_{\mathrm{i}}+{\upbeta }_{15}{\mathrm{Other}-\mathrm{ethnic}}_{\mathrm{i}}\\ &\quad+\sum_{\mathrm{j}=2}^{4}{{\upgamma }_{\mathrm{j}}\mathrm{Quartile}}_{\mathrm{j}}*{\mathrm{Bangladeshi}}_{\mathrm{i}}+ \sum_{\mathrm{j}=2}^{4}{{\mathrm{\varnothing }}_{\mathrm{j}}\mathrm{Quartile}}_{\mathrm{j}}*{\mathrm{Indian}}_{\mathrm{i}}+\sum_{\mathrm{j}=2}^{4}{{\upmu }_{\mathrm{j}}\mathrm{Quartile}}_{\mathrm{j}}*{\mathrm{Pakistani}}_{\mathrm{i}}\\ &\quad++\sum_{\mathrm{j}=2}^{4}{{\uptau }_{\mathrm{j}}\mathrm{Quartile}}_{\mathrm{j}}*{\mathrm{Arab}}_{\mathrm{i}}+\sum_{\mathrm{j}=2}^{4}{{\mathrm{\vartheta }}_{\mathrm{j}}\mathrm{Quartile}}_{\mathrm{j}}*{\mathrm{Chinese}}_{\mathrm{i}}+\sum_{\mathrm{j}=2}^{4}{{\upeta }_{\mathrm{j}}\mathrm{Quartile}}_{\mathrm{j}}*\mathrm{Asian}-{\mathrm{Other}}_{\mathrm{i}}\\ &\quad+\sum_{\mathrm{j}=2}^{4}{{\updelta }_{\mathrm{j}}\mathrm{Quartile}}_{\mathrm{j}}*\mathrm{Black}-{\mathrm{African}}_{\mathrm{i}}+\sum_{\mathrm{j}=2}^{4}{{\uptheta }_{\mathrm{j}}\mathrm{Quartile}}_{\mathrm{j}}*\mathrm{Black}-{\mathrm{Carribean}}_{\mathrm{i}}\\ &\quad+\sum_{\mathrm{j}=2}^{4}{{\uplambda }_{\mathrm{j}}\mathrm{Quartile}}_{\mathrm{j}}*\mathrm{Black}-{\mathrm{Other}}_{\mathrm{i}}+\sum_{\mathrm{j}=2}^{4}{{\uppsi }_{\mathrm{j}}\mathrm{Quartile}}_{\mathrm{j}}*{\mathrm{Mixed}}_{\mathrm{i}}\\ &\quad+\sum_{\mathrm{j}=2}^{4}{{\upomega }_{\mathrm{j}}\mathrm{Quartile}}_{\mathrm{j}}*{\mathrm{Other}-\mathrm{ethnic}}_{\mathrm{i}}+{\mathrm{\vartheta }{\mathrm{pop}-\mathrm{density}}_{i}+\Psi {\mathrm{edu}-\mathrm{status}}_{i }}_{\mathrm{i}}+{\mathrm{e}}_{\mathrm{i}}\end{aligned} $$

where $\mathrm{W}$ denotes a spatial contiguity matrix, and $\mathrm{e}$ the i.i.d disturbance term (Drukker et al.^23^).

We also estimated a spatial regression model without the interaction terms. The estimated coefficient on the spatial lag of age-adjusted mortality is 0.407 (CI 0.292–0.522, *p* < 0.001), indicating positive correlation between the age-adjusted mortality in one LAD and the age-adjusted mortality in a neighbouring LAD. The total effect remained qualitatively the same as the model with interaction terms as reported in the paper.

## Results

Table 1 captures the variation in means across some key variables viz. age standardized COVID-19 mortality, the index of multiple deprivation (IMD) and the dis-aggregated ethnicity profiles. The overall average age-standardized mortality was 35.501 per 100,000 people with a standard deviation of 24.858. However, the average age-standardized mortality for the most deprived areas (Quartile 4) was 47.21 per 100,000 people compared to 27.727 per 100,000 people for the least deprived areas (Quartile 1). The percentage of ‘Black-African’, ‘Black-Caribbean’, ‘Black-Other’, ‘Bangladeshi’ and ‘Pakistani’ population increased across the quartiles of Index of multiple deprivation whereas for the ‘White’, the trend reverses. Percentage of ‘Indian’, ‘Chinese’ and ‘Asian-Other’ population increases for higher quartiles (except for Quartile 3).

### Regression results

The results are reported in Table 2. The coefficient associated with each ethnicity interacted with deprivation quartile in Table 2 reflects the aggregate effect of being in the particular deprivation quartile and belonging to the corresponding ethnicity.

**Table 2:** Age adjusted mortality rates and association between ethnicity and index of multiple deprivation, linear regression model: coefficients (95% confidence interval, *P* value).

Variable	Coefficient	(Confidence interval), *p* value
Quartile dummy 1	–	–
Quartile dummy 2	− 2.108	(− 16.570 to 12.353), *p* = 0.774
Quartile dummy 3	5.977	(− 7.203 to 19.156), *p* = 0.373
Quartile dummy 4	16.270	(2.355 to 30.185), *p* = 0.022
White	–	–
Black-African in 2nd quartile dummy	4.438	(− 0.096 to 8.972), *p* = 0.055
Black-African in 3rd quartile dummy	2.925	(1.102 to 4.748), *p* = 0.002
Black-African in 4th quartile dummy	2.861	(1.080 to 4.642), *p* = 0.002
Black-Caribbean in 2nd quartile dummy	12.166	(4.209 to 20.123), *p* = 0.003
Black-Caribbean in 3rd quartile dummy	7.276	(2.224 to 12.328), *p* = 0.005
Black-Caribbean in 4th quartile dummy	9.655	(5.248 to 14.061), *p* < 0.001
Black-Other in 2nd quartile Dummy	− 33.252	(− 49.193 to − 17.311), *p* < 0.001
Black-Other in 3rd quartile dummy	− 6.861	(− 19.842 to 6.121), *p* = 0.299
Black-Other in 4th quartile dummy	− 7.882	(− 20.924 to 5.160), *p* = 0.235
Bangladeshi in 2nd quartile dummy	− 16.183	(− 22.999 to − 9.366), *p* < 0.001
Bangladeshi in 3rd quartile dummy	− 3.645	(− 7.277 to − 0.013), *p* = 0.049
Bangladeshi in 4th quartile dummy	1.952	(1.144 to 2.760), *p* < 0.001
Indian in 2nd quartile dummy	5.122	(3.114 to 7.129), *p* < 0.001
Indian in 3rd quartile dummy	1.604	(0.037 to 3.171), *p* = 0.045
Indian in 4th quartile dummy	0.225	(− 0.990 to 1.441), *p* = 0.715
Pakistani in 2nd quartile dummy	1.225	(− 0.899 to 3.348), *p* = 0.257
Pakistani in 3rd quartile dummy	0.230	(− 1.190 to 1.650), *p* = 0.750
Pakistani in 4th quartile dummy	0.487	(− 0.366 to 1.339), *p* = 0.262
Chinese in 2nd quartile dummy	0.034	(− 8.790 to 8.858), *p* = 0.994
Chinese in 3rd quartile dummy	6.242	(− 6.134 to 18.619), *p* = 0.322
Chinese in 4th quartile dummy	− 4.708	(− 12.532 to 3.116), *p* = 0.237
Asian-Other in 2nd quartile dummy	− 1.922	(− 3.731 to − 0.112), *p* = 0.038
Asian-Other in 3rd quartile dummy	− 0.362	(− 3.461 to 2.738), *p* = 0.818
Asian-other in 4th quartile dummy	− 3.181	(− 11.553 to 5.192), *p* = 0.455
Arab in 2nd quartile dummy	− 37.071	(− 76.589 to 2.446), *p* = 0.066
Arab in 3rd quartile dummy	− 0.096	(− 3.206 to 3.014), *p* = 0.952
Arab in 4th quartile dummy	16.539	(− 0.659 to 33.736), *p* = 0.059
Mixed in 2nd quartile dummy	3.408	(− 5.701 to 12.517), *p* = 0.462
Mixed in 3rd quartile dummy	− 4.008	(− 9.237 to 1.220), *p* = 0.132
Mixed in 4th quartile dummy	− 6.836	(− 11.473 to − 2.199), *p* = 0.004
Other ethnic group in 2nd quartile dummy	23.201	(10.627 to 35.775), *p* < 0.001
Other ethnic group in 3rd quartile dummy	3.163	(− 3.582 to 9.908), *p* = 0.357
Other ethnic group in 4th quartile dummy	6.422	(2.266 to 10.579), *p* = 0.003
Proportion of people with A-level	− 30.625	(− 75.139 to 13.888), *p* = 0.177
Population density	0.275	(0.111 to 0.439), *p* = 0.001
No. of observations	315	

White ethnicity is being used as the base category.

It is clear that even after controlling for ethnicity, deprivation matters: most deprived LADs also exhibited significantly larger mortality compared to the affluent quartile (see ‘Quartile Dummy 4’ in Table 2). Interaction of economic deprivation with dis-aggregated ethnicity confirmed considerable heterogeneity within the BAME community. For example, a 1 percentage point increase in ‘Black-African’ population in the poorest LADs (Quartile 4) increased mortality rate by 2.861 per 100,000 population (CI 1.080–4.642, *p* = 0.002) and the corresponding increase for the ‘Black-Caribbean’ population in the fourth Quartile is 9.655 (CI 5.248–14.061, *p* < 0.001). The results were not as clear for the ‘Bangladeshi’ community, whereby the second and third Quartile effect was negative. Whereas a 1% point increase in ‘Bangladeshi’ community in the poorest LADs (Quartile 4) significantly increased COVID-19 mortality by 1.952 per 100,000 population (CI 1.144–2.760, *p* < 0.001) compared to the ‘White’ community. Similar positive significant impact was also established for the ‘Indian’ in the second and in the third Quartiles. ‘Other ethnic group’ across all the three deprivation quartiles record a significantly higher age-adjusted mortality rate compared to the ‘White’ group. A unit increase in population density (see *pop-density* in Table 2) increased mortality by 0.275 per 100,000 population (CI 0.111–0.439, *p* = 0.001).

### Spatial regression results

The spatial regression results based on dis-aggregated ethnicity are reported in Table 3. The estimated coefficient on the spatial lag of age-adjusted mortality $(\widehat{\phi })$ was 0.462 (CI 0.355–0.569, *p* < 0.001), indicating positive correlation between the age-adjusted mortality in one LAD and the age-adjusted mortality in a neighbouring LAD.

**Table 3:** Marginal effects of ethnicity on age adjusted mortality, spatial regression model: coefficients (95% confidence interval, *P *value).

Ethnicity	Median versus 25th percentile	75th percentile versus 25th percentile	75th percentile versus median
**Deprivation Quartile 2**			
Black-African	2.294 (− 0.255 to 4.842), *p* = 0.078	8.158 (− 0.906 to 17.221), *p* = 0.078	5.864 (− 0.651 to 12.379), *p* = 0.078
Black-Caribbean	2.096 (− 0.120 to 4.312), *p* = 0.064	8.111 (− 0.464 to 16.687), *p* = 0.064	6.015 (− 0.344 to 12.374), *p* = 0.064
Bangladeshi	− 1.995 (− 2.914 to − 1.075), *p* < 0.001	− 8.184 (− 11.955 to − 4.413), *p* < 0.001	− 6.189 (− 9.042 to − 3.337), *p* < 0.001
Indian	1.819 (0.682 to 2.955), *p* = 0.002	8.191 (3.072 to 13.311), *p* = 0.002	6.373 (2.390 to 10.355), *p* = 0.002
Pakistani	0.713 (0.098 to 1.329), *p* = 0.023	4.443 (0.608 to 8.278), *p* = 0.023	3.730 (0.510 to 6.949), *p* = 0.023
**Deprivation Quartile 3**			
Black-African	2.419 (0.027 to 4.810), *p* = 0.047	8.603 (0.097 to 17.109), *p* = 0.047	6.184 (0.070 to 12.299), *p* = 0.047
Black-Caribbean	3.092 (1.105 to 5.080), *p* = 0.002	11.966 (4.274 to 19.658), *p* = 0.002	8.874 (3.170 to 14.578), *p* = 0.002
Bangladeshi	− 1.220 (− 1.962 to − 0.477), *p* = 0.001	− 5.004 (− 8.049 to − 1.959), *p* = 0.001	− 3.784 (− 6.087 to − 1.481), *p* = 0.001
Indian	1.344 (0.130 to 2.557), *p* = 0.030	6.052 (0.588 to 11.516), *p* = 0.030	4.708 (0.457 to 8.959), *p* = 0.030
Pakistani	0.045 (− 0.379 to 0.469), *p* = 0.836	0.279 (− 2.363 to 2.922), *p* = 0.836	0.235 (− 1.984 to 2.453), *p* = 0.836
**Deprivation Quartile 4**			
Black-African	1.795 (− 0.597 to 4.187), *p* = 0.141	6.385 (− 2.122 to 14.893), *p* = 0.141	4.590 (− 1.526 to 10.706), *p* = 0.141
Black-Caribbean	3.462 (1.577 to 5.348), *p* < 0.001	13.398 (6.102 to 20.695), *p* < 0.001	9.936 (4.525 to 15.347), *p* < 0.001
Bangladeshi	− 0.557 (− 1.098 to − 0.017), *p* = 0.043	− 2.287 (− 4.505 to − 0.069), *p* = 0.043	− 1.730 (− 3.407 to − 0.052), *p* = 0.043
Indian	0.629 (− 0.272 to 1.529), *p* = 0.171	2.833 (− 1.223 to 6.889), *p* = 0.171	2.204 (− 0.952 to 5.359), *p* = 0.171
Pakistani	0.142 (− 0.214 to 0.498), *p* = 0.434	0.884 (− 1.332 to 3.100), *p* = 0.434	0.742 (− 1.118 to 2.603), *p* = 0.434

Instead of regression coefficients, we focused on the obtained marginal effects for select ethnicity profile. The results are reported in Table 3 for five ethnicity groups: ‘Black-African’, ‘Black-Caribbean’, ‘Bangladeshi’, ‘Indian’ and ‘Pakistani’. Table 3 looks at the obtained results in a more disaggregated manner. Here we conducted comparison of three scenarios for each of the deprivation quartile: keeping the ethnicity at the 25th percentile, at the median and at the 75th percentile within each deprivation category. The results show that if the ‘Black-Caribbean’ population stood at the 75th percentile in comparison to the 25th percentile, the mortality rate would increase by 8.111 (CI − 0.464–16.687, *p* = 0.064), 11.966 (CI 4.274–19.658, *p* = 0.002), and 13.398 (CI 6.102–20.695, *p* < 0.001) for the ‘Quartile dummy’ 2, 3 and 4 respectively. The ‘Black-Caribbean’ showed an increase of 2.096 (CI − 0.120–4.312, *p* = 0.064), 3.092 (CI 1.105–5.080, *p* = 0.002) and 3.462 (CI 1.577–5.348, *p* < 0.001) when we compared the median in comparison to the 25th percentile. The other statistically significant increase was observed for the ‘Black-African’, and ‘Indian’ for the second and third Quartile of deprivation and for ‘Pakistani’ only for the second Quartile of deprivation. Contrary, to previous literature, ‘Bangladeshi’ had a significantly lower age-adjusted mortality with deterioration in deprivation status. For example, if the ‘Bangladeshi’ population stood at the 75th percentile in comparison to the 25th percentile, the mortality rate would decrease by 8.184 (CI − 11.955 to − 4.413, *p* < 0.001), 5.004 (CI − 8.049 to − 1.959, *p* < 0.001) and 2.287 (CI − 4.505 to − 0.069, *p* = 0.043) for the ‘Quartile dummy’ 2, 3 and 4 respectively.

## Discussion

We found that COVID-19 mortality disproportionately affects the local areas with an over-representation of individuals who are relatively socio-economically deprived and also belong to ethnic minority particularly the ‘Black-Caribbean’ and ‘Black-African’. Linear regression estimates showed that considering ‘South-Asian’ community as one homogeneous entity can be misleading because there was considerable discrepancy among the ethnic subgroups (‘Bangladeshi’, ‘Indian’ and ‘Pakistani’) across the different deprivation quartiles. Impact of living in a particular LAD (neighbourhood effect) is also confirmed by positive significant impact of population density within each LAD on the age adjusted COVID-19 mortality. The spatial regression results suggest a strong spillover effect in the disease burden. The spatial regression estimates also corroborates the higher risks of particularly the ‘Black-Caribbean’—for each deprivation quartile, age adjusted mortality rate became significantly higher if the percentage of ‘Black Caribbean’ increased in that LAD. A similar pattern was also seen for the ‘Black-African’, ‘Indian’ in second and third Quartile, and for ‘Pakistani’ in the second Quartile. Amongst the ‘South-Asian’ population, the ‘Bangladeshi’ behaves differently.

Pareek et al.^7^ have rightly called for an urgent public health approach to understand the differential effect of ethnicity and understand the interplay of ethnicity with several factors. Apart from the inherent genetic disposition, our analysis sheds light on deprivation e.g. in inequality in access to resources for the higher prevalence of mortality in certain segments of the population. Our analysis findings largely correlate with existing evidence suggesting that economic deprivation with dis-aggregated ethnicity exhibits considerable heterogeneity within the BAME community. Consistent with ONS^4^ and Apea et al.^24^ individuals from ‘Black-African’ and ‘Black-Caribbean’ community and who are also economically more deprived record a significantly higher age-adjusted COVID-19 mortality compared to the ‘White’.

However, our results are particularly interesting when we disaggregate by ethnic group. Our results are in contrast to previous literature^10^ whereby no consistent pattern emerges when we compare the combined effect of ethnicity and economic deprivation across the different ‘South Asian’ community profile. Impact for the ‘Bangladeshi’ is negative and significant contrary to previous findings^10^. While we cannot fully explain this, many of these areas may have other compensating factors such as higher social capital and other LAD specific factors that we cannot observe in the data. An empirical analysis of how such differences in behavioural norms and choices across ethnicities have impacted their risk of contracting the deadly virus is left for future investigation. Despite some similarities with pre-existing literature, our results suggest a differential public health approach would be warranted to capture the nuances within the different ethnic minority groups.

The most important limitation with this type of study is the possibility of ecological fallacy, whereby the effect seen at a LAD level may not be generalisable to individuals in the region. Another important limitation is that in this study we were unable to adjust for important co-variates such as occupation and comorbidity status all of which may be risk factors for COVID-19^6^ although we were able to account for population density and educational attainment. It is important to highlight that ethnic groups do have differing occupational profiles which may have an impact on their COVID-19 risk. Of the total employed Black population predominant share belonged to ‘Caring Personal Service Occupations’ (15.4%), ‘Elementary Administration and Service Occupations’ (14.3%) and in ‘Health Professionals’ (9%). In comparison for the employed White, there was a more uniform distribution across different occupations (for example while 6.9% were working in ‘Caring Personal Service Occupations’ a very large proportion (8.0%) were also ‘Corporate Managers and Directors’)^25^. The largest share of employed Asian is in ‘Elementary Administration and Service Occupations’ (50.5%). If we look at NHS per se, the latest data reveals that “Asian people made up a higher percentage of medical staff (at 29.7%) than non-medical staff (at 8.0%)”^26^. These occupational roles may put BAME communities at a greater risk of COVID-19 and may make it more difficult to practice social distancing^27^. Additionally, it is clear that BAME communities have a higher level of general comorbidity which may pose as an independent risk for COVID-19^28^.

In conclusion, our results clearly show that deprivation and its interaction with ethnicity play an important role in explaining COVID-19 mortality. The presence of spatial effects and spillover suggest family structures and social networks play an important role too. Social interactions between people across neighbouring regions can also spread the disease.

## Supplementary Information

Supplementary Information.

## Data Availability

The data used in this study was open source provided by the Local Authority. Any requests for the extracts used during this study period can be made to the corresponding author.
